# Healthy foods, healthy sales? Cross-category spillover effects of a reward program promoting sales of fruit and vegetables

**DOI:** 10.23889/ijpds.v8i3.2270

**Published:** 2023-09-11

**Authors:** Luca Panzone, Barbara Tocco, Ruzica Brecic, Matthew Gorton

**Affiliations:** 1 Newcastle University; 2 University of Zagreb

## Introduction & Background

Globally, consumption of Fruit and Vegetables (F&V) remains below nutritional guidelines. Marketing healthy products can be complex for retailers, and a key challenge is the design of strategies that benefit retailers, e.g., through improved loyalty, and deliver progress on societal goals.

## Objectives & Approach

This study evaluates a point-plus-cash frequency reward program where participants received points by purchasing selected F&V, redeemable against a reward (plush toys in the shape of F&V). We estimate the impact of the program using a difference-in-difference-in-difference model, which compares expenditures in several categories before, during, and after the promotional period, across two different years, and separately for consumers who redeemed a reward and those who did not. Identification includes weighting for the propensity scores, and using an instrumental variable approach.

## Relevance to Digital Footprints

The data refers to grocery expenditure in five categories in the focal retailer for over 268,000 consumers, over 27 weeks for 2 years, as recorded through their loyalty card.

## Results

The reward program significantly increased expenditures in F&V in the focal retailer during the promotional period. However, results differed depending on reward redemption. For reward-redeemers, the program increased expenditures in F&V as well as in other food categories, an effect that persisted – at a declining rate – after the program stopped. The program had a short-lived effect on non-rewards redeemers, who only increased F&V expenditures during the promotional period.

## Conclusions & Implications

Results indicate that a loyalty program promoting sales of F&V can create win-win benefits to both society and the retailer: it increases expenditures on healthy foods (F&V), while improving overall loyalty (i.e., expenditures) to the retailer.

